# Study on the Stability of Complex Networks in the Stock Markets of Key Industries in China

**DOI:** 10.3390/e26070569

**Published:** 2024-06-30

**Authors:** Zinuoqi Wang, Guofeng Zhang, Xiaojing Ma, Ruixian Wang

**Affiliations:** 1 School of Economics, Hebei GEO University, Shijiazhuang 050031, China; zinuoqiwang@gmail.com (Z.W.); ruixianw6@gmail.com (R.W.); 2 Research Base for Scientific-Technological Innovation and Regional Economic Sustainable Development of Hebei Province, Hebei GEO University, Shijiazhuang 050031, China; 3 Natural Resource Asset Capital Research Center, Hebei GEO University, Shijiazhuang 050031, China; 4 School of Earth Sciences, Hebei GEO University, Shijiazhuang 050031, China; maxiaojing@hgu.edu.cn

**Keywords:** stock market, complex network, stability, systematic risk entropy

## Abstract

Investigating the significant “roles” within financial complex networks and their stability is of great importance for preventing financial risks. On one hand, this paper initially constructs a complex network model of the stock market based on mutual information theory and threshold methods, combined with the closing price returns of stocks. It then analyzes the basic topological characteristics of this network and examines its stability under random and targeted attacks by varying the threshold values. On the other hand, using systemic risk entropy as a metric to quantify the stability of the stock market, this paper validates the impact of the COVID-19 pandemic as a widespread, unexpected event on network stability. The research results indicate that this complex network exhibits small-world characteristics but cannot be strictly classified as a scale-free network. In this network, key roles are played by the industrial sector, media and information services, pharmaceuticals and healthcare, transportation, and utilities. Upon reducing the threshold, the network’s resilience to random attacks is correspondingly strengthened. Dynamically, from 2000 to 2022, systemic risk in significant industrial share markets significantly increased. From a static perspective, the period around 2019, affected by the COVID-19 pandemic, experienced the most drastic fluctuations. Compared to the year 2000, systemic risk entropy in 2022 increased nearly sixtyfold, further indicating an increasing instability within this complex network.

## 1. Introduction

In the past few years, China’s economic development has shown vigorous vitality in the complex and changing international environment, continuously achieving a virtuous cycle of domestic and international economic and trade activities. This is inseparably linked to the economic resilience provided by China’s stable economic network. Meanwhile, the unstoppable wave of economic globalization has made the interdependence and interconnectedness between international financial markets increasingly apparent [1,2,3]. Through economic activities such as import and export trade [4,5] and cross-border investment [6], the financial networks constructed by countries not only supply fresh blood to national economic development but also provide channels for the contagion of systemic financial risks. The COVID-19 pandemic, as an unexpected exogenous event, significantly impacted global trade and investment activities, further leading to a subdued overall performance of financial markets. In April 2020, the three major US stock indices—the Dow Jones Industrial, S&P 500, and NASDAQ—experienced severe declines, falling by 20.5%, 17.6%, and 11.8% from the beginning of the year, respectively. The negative repercussions of the COVID-19 pandemic on international capital marketplaces and major commodity markets, including crude oil, have surpassed those experienced during the 2008 global financial meltdown [7]. China’s mainland A-share market also experienced unprecedented violent fluctuations, with over 3000 stocks hitting their down-limits. This underscores the importance for countries to always guard against systemic financial risks and maintain financial market stability, as both are complementary and crucial for achieving sustainable and healthy economic development.

Overall, the academic community’s theoretical guidance on financial market issues has transitioned from neoclassical financial theory to behavioral finance and now to the current complexity science. This shift employs new research paradigms, ideological frameworks, and knowledge systems to reveal the complex, dynamic, and non-equilibrium structure and inherent laws of financial markets, pushing the research towards directions of greater universal significance and closer alignment with reality.

Numerous scholars have employed a variety of methods to analyze factors causing fluctuations in the equity marketplace and to measure indicators of whether the market is in a stable state, thereby providing indirect evidence for the necessity of researching stock market stability. Diebold and Yilmaz, through empirical methods, unveiled the connection between macroeconomic fundamentals’ fluctuations and stock market stability, a connection that might be overlooked by mere time-series analysis [8]. Son et al. utilized a statistical model centered around the Stock Market Stability Index (SMSI) to monitor the robustness of the South Korean share market; should this model prove ineffective, it would signify that the market is in a state of instability [9]. Chen et al. found that stock index futures trading significantly improves the stability of the Chinese share market if it is combined with a panel data policy evaluation method [10]. Resta combined self-organizing maps (SOMs) and minimum spanning trees (MSTs) to study market conditions characterized by varying degrees of stability, ultimately achieving early risk warning [11].

Scholars starting from the behavioral finance theory have focused on investor psychology and behavioral traits as key factors influencing financial market stability. Phenomena such as herd behavior and positive feedback serve as signals that market stability is under threat [12]. Chiang and other researchers discovered proof of herding behavior in developed equity marketplaces (excluding the United States) and Asian markets [13]. Additional studies uncovered evidence of herding tendencies among investors in China’s A-share market, contingent upon the variability of stock returns within the lower quantiles as analyzed by quantile regression [14]. Gardini et al., by constructing a behavioral stock market model, discovered that the existence of emotional bargainers could impair the stability of the share marketplace [15]. Spelta et al. have identified the onset of market instability by measuring the strength of self-organizing activities that arise due to the synchronicity in stock returns [12].

Currently, scholars are increasingly focusing on exploring issues of financial market stability through complex networks [16,17]. Billio et al. investigated the interconnectedness of international financial markets using principal component analysis and Granger causality analysis, suggesting that complex networks can measure, detect, and prevent the destruction of financial crises, thus maintaining stability [18]. Heiberger studied the stability of the S&P 500 market during financial crises using a winner-takes-all principle, finding that the market forms a concentrated topological structure to adapt to crises [19]. Mantegna initially employed the minimum spanning tree approach to model the stock market and reveal its general hierarchy and then used the price fluctuations of American stocks to construct a hierarchical network and replicate the market’s topological properties, aiming to capture essential market information while reducing market complexity as much as possible [20]. Zhang and Zhuang, by constructing a system of the Chinese share market, discovered a Granger causal relevance between share marketplace volatility and the network steadiness parameter, with system connectivity and clustering coefficient negatively correlated with equity market volatility [21]. Huang et al. used financial categorization and threshold methods to construct a network and analyze the stability of China’s equity marketplace, believing their conclusions could also provide effective recommendations for securities investment and risk management [22].

In the broad spectrum of scholarly research, volatility is synonymous with risk and uncertainty [23]. Another approach to analyzing economic or financial issues, such as stock market instability, involves applying the concept of entropy from physics. Gradojevic and Caric have discovered that the analysis of entropy values is effective for predictive marketplace risk management, with the predictability level intimately associated with the entropy type and the characteristics of the base signals [24].

In summary, scholars have witnessed an evolution in the methodologies for researching equity market stability, transitioning from econometric to complex network analyses. This shift has allowed for a comprehensive capture of the dynamic complexities within market movements, though the perspective has predominantly been macroscopic. In light of this, the marginal contribution of this paper lies in adopting a “top-down” research perspective, focusing on China’s key industries, and employing mutual information and threshold methods to construct a complex network model of the stock market for analyzing its topological structure. This approach further facilitates the identification of major industries within the complex network model and assesses network stability under random and targeted attack strategies. Additionally, the paper utilizes systemic risk entropy to quantify network stability, confirming the impact of the COVID-19 pandemic as a widespread, unexpected event on network stability. It specifically focuses on representative enterprises to target business dynamics proactively, with the aim of preemptively managing risks and minimizing losses.

The marginal contribution of this paper lies in the adoption of a “top-down” research perspective, focusing on key industries in China. By using mutual information and the threshold method, we construct a complex network model of the stock market to analyze its topological characteristics. To quantify the stability of the network, this paper introduces the systemic risk entropy index, which can measure the robustness of the complex network when facing shocks. This index offers a new perspective for risk management and predicts risk variations under different market conditions. Additionally, the conclusion and recommendations of this paper emphasize the need to pay special attention to the operational dynamics of representative companies. By proactively identifying and monitoring the risk factors of these key enterprises, we can provide targeted risk management advice to both companies and investors. This helps them to anticipate risks and mitigate potential losses during market fluctuations.

## 2. Data Material and Methods

### 2.1. Data Selection

This paper focuses on key industry sectors within the securities trading market that are large in scale, well developed, and highly correlated with the national economy. This study selects data from the period of 1 January 2000 to 24 September 2020, during normal trading periods (excluding weekends and holidays). To minimize the impact of incomplete data on the network stability analysis, stocks with long suspension periods and incomplete data were excluded. Ultimately, a sample of 2488 stocks collected from the Shanghai and Shenzhen Stock Exchanges was used to construct a complex financial securities network, covering 11 major industries: real estate, industry, building materials, transportation, finance, media information services, energy and environment, consumer goods, and pharmaceuticals and healthcare.

It is important to note that this study chooses daily data over intraday data mainly for the following reasons. First, intraday data may contain a significant amount of market noise, which can affect the calculation of mutual information. Daily data, being relatively more stable, can reduce the interference from short-term fluctuations, thereby more accurately reflecting the long-term relationships between stocks. Second, this study aims to explore the long-term stability and overall behavior patterns of the stock market. Daily data are more suitable for capturing long-term trends and the overall structure of the market.

Each stock is treated as a point within the web, and the correlation between any two stocks is considered an edge in the system. The effective trading day price series of a stock is denoted as $\left\{p_{i}\left(1\right),p_{i}\left(2\right),\left.p_{i}\left(N_{i}\right)\right\}\right.$, where $N_{i}$ represents the number of effective trading days for stock *i*, and $p_{i}\left(t\right)$ represents the closing price of stock *i* on the *t*-th effective trading day. The daily closing price return rate of a stock is defined as follows:

(1) $$R_{i}(t)=\ln p_{i}(t)-\ln p_{i}(t-1)$$

### 2.2. Construction of Matrix Based on Mutual Information

Mutual information is a measure of the mutual dependence between two random variables. It quantifies the extent to which the information of one variable can be obtained through another variable.

In the financial market, the overall situation of stock prices exhibits volatility, uncertainty, and non-linearity, yet changes in different stock prices share certain correlations. Mutual information theory, which measures distance without relying on theoretical and statistical distributions of data, is a non-parametric method. This method remains effective in measuring the dependency between financial data even in the face of drastic market fluctuations. Simultaneously, Guo X et al., in their study on the relationships between stocks traded on the Shanghai Stock Exchange, evaluated the effectiveness of measuring stock relationships by comparing correlation coefficients and mutual information. They found that in a stock market where stock prices can experience significant fluctuations, mutual information is a more effective method than correlation coefficients for measuring stock relationships [25]. Sharma C and Habib A, by using mutual information to study the nonlinear interactions in high-frequency data of the Indian stock market, effectively revealed the network of stock returns [26]. The mutual information between discrete and continuous random variables can be defined as follows:

(2) $$I(X,Y)=\sum_{x\in X,y\in Y}p(x,y)\log\frac{p(x,y)}{p_{X}(x)p_{Y}(y)}$$

(3) $$I(X,Y)=\iint_{X,Y}f(x,y)\log\frac{f_{X,Y}(x,y)}{f_{X}(x)f_{Y}(y)}dxdy$$

Among them, *P*(*x*,*y*) is the joint probability of *X* and *Y*, *P_X_*(*x*) and *P_Y_*(*y*) are the marginal probabilities of *X* and *Y*, respectively, *f*(*x*,*y*) is the joint probability density function of *X* and *Y*, and *f_X_*(*x*) and *f_Y_*(*y*) are the marginal probability density functions of *X* and *Y*, respectively. The notation (*X*,*Y*) is used to indicate that *X* and *Y* are discrete variables. This definition indicates how much information about *X* can be known if the value of the variable *Y* is already known. From our definition, we can see that if *X* and *Y* are independent, i.e., *f*(*x*,*y*) = *f_X_*(*x*)*f_Y_*(*y*), then the mutual information *I*(*X*,*Y*) is zero. In other words, if the value of *Y* is known, the amount of information known about *X* is zero. This is consistent with the concept of independence. Based on the formula above, the mutual information between every pair of stocks is calculated, and a mutual information matrix is constructed.

### 2.3. Probability Density Estimation of the Complex Network of Financial Stocks

**Figure 1:**
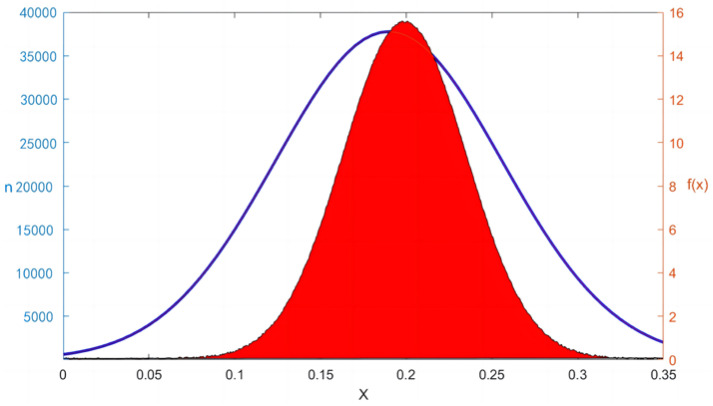
Probability density distribution of mutual information among stocks.

Based on the closing prices and using the formula for return rates, the relative daily return rates for each stock were calculated. Subsequently, the mutual information formula was applied to compute the mutual information matrix of the pairwise stocks’ relative daily return rates, further deriving the corresponding probability density distribution, as shown in Figure 1.

In Figure 1, the *X*-axis represents different mutual information value intervals, used to measure the mutual dependence between stock pairs. The left vertical axis n shows the sample count corresponding to different mutual information values *X*, and the right vertical axis displays the probability density *f*(*x*) of the mutual information values. From this figure, it can be observed that the distribution of mutual information among the stocks is generally close to a normal distribution. Through fitting with Matlab 2023a, the parameters for the normal distribution expression based on mutual information were found to be μ = 0.183, σ = 0.048.

### 2.4. Calculation of Mutual Information in the Complex Network of Financial Stocks

The first step involves calculating the daily closing price return rate of stocks according to Equation (1). The second step uses an equidistant algorithm to calculate the information entropy and mutual information between stock *X* and stock *Y*. It satisfies the following three conditions: First, mutual information itself is non-negative, i.e., *I*(*X*;*Y*) ≥ 0, which meets the non-negativity requirement of a distance measure. When *X* and *Y* are completely independent, the mutual information is zero, indicating no shared information between them. Second, mutual information is symmetric, *I*(*X*;*Y*) = *I*(*Y*;*X*), and the distance based on mutual information also maintains this symmetry.

Finally, a transformed distance based on mutual information is defined as *d*(*X*,*Y*) = √1 − exp(−2·*I*(*X*;*Y*)). Initially, the number of intervals is calculated according to Equation (4), where *m* is the number of intervals, and *n* is the sample size.

(4) $$m=1.87\times {\left(n-1\right)}^{2/5}$$

Subsequently, the interval width is calculated according to Equation (5). For stock *X*, its interval width can be expressed as follows:

(5) $$h_{x}=\left(\max x-\min x\right)/m$$

where max *x* is the maximum value in the series of stock *X*, and min *x* is the minimum value. The final step is the estimation of the probability density function. For two time series *X_i_* and *Y_i_* with a sample size of *n*, max *x* and min *x*, and max *y* and min *y*, respectively, represent the maximum and minimum values of the series *X_i_* and *Y_i_*. Dividing the intervals [min *x*, max *x*] and [min *y*, max *y*] into *m* parts, denoted as A_1_, A_2_, …, A*_j_* and B_1_, B_2_, …, B*_j_*, the sample volume falling within intervals A*_j_* and B*_j_* for time series *X_i_* and *Y_i_* are, respectively, denoted as *p_i_*, *q_i_*, with the sample size denoted as *ω_ij_*. Thus, the probability density function and joint probability density function for *X_i_* and *Y_i_* can be represented as follows:

(6) $$\hat{f}(x)=\frac{p_{i}}{{nh}_{x}}(i=1,2,\ldots \ldots ,m)$$

(7) $$\hat{f}(y)=\frac{q_{i}}{{nh}_{y}}(i=1,2,\ldots \ldots ,n)$$

(8) $$\hat{f}(x,y)=\frac{\omega_{ij}}{nh_{x}h_{y}}(i=1,2,\ldots \ldots ,n)$$

Following this, information entropy and mutual information are calculated. Information entropy for *X_i_* and *Y_i_* is computed according to Equations (9) and (10), respectively, and mutual information between the two variables is calculated using Equation (11). Finally, mutual information is standardized in accordance with Equation (12):

(9) $$H(X)=-\int_{X}f(x)\log f(x)dx$$

(10) $$H(X,Y)=-\iint_{X,Y}f(x,y)\log f(x,y)dxdy$$

(11) I(X,Y)=H(X)+H(Y)−H(X,Y)

(12) $$\mathrm{NMI}=\frac{I(X,Y)}{\sqrt{H(X)H(Y)}}$$

### 2.5. Selection and Optimization of the Effective Threshold Range

By selecting an appropriate threshold using the threshold method, redundant information can be reduced, making the network more streamlined. For any nodes *i* and *j*, if the normalized mutual information (NMI*_ij_*) between these two nodes is greater than or equal to the determined threshold *θ* ∈ [−1,1], an edge is added between nodes *i* and *j*; otherwise, no edge is added. The setting of the threshold determines whether the variables can participate in the construction of the network. This paper optimizes the threshold range by observing changes in the points within the largest interconnected subgraph as the threshold varies. Based on a determined effective threshold range, a more effective threshold range and a reasonable threshold are identified by analyzing the inflection points on the curve that depicts the change in the number of points in the largest connected subgraph with increasing threshold values.

### 2.6. Construction of the Complex Network of the Share Market

Before investigating the complex network of financial shares, it is necessary to define the nodes and edges of this network. Standardize the daily return data of the stocks, then calculate the mutual information between each pair of stocks to generate the mutual information matrix *I*, where *I_ij_* represents the mutual information value between stock *i* and stock *j*. Based on the selected threshold *θ*, if *I_ij_* is greater than or equal to *θ*, set the corresponding position in the adjacency matrix to 1; otherwise, set it to 0.

### 2.7. Two Different Network Attack Methods Based on Node Removal

In the complex network of financial shares, the degree of a node represents its relationship with other nodes. Therefore, the higher the degree value, the closer its relationship with other nodes, or in other words, the greater its influence. When studying the stability of complex networks, there are generally two methods based on node removal: (1) Random attack: some nodes and all their connected edges in the complex network of financial securities are deleted entirely at random. (2) Intentional attack: nodes in the complex network of financial securities are removed in order of importance from highest to lowest, thus carrying out an intentional attack on the complex network of the stock market.

### 2.8. Calculation of Systematic Risk Entropy

There is a close relationship between the stability of intricate networks of share marketplaces and the entropy of systemic risk, which together form an important framework for understanding and assessing the stability and risk level of complex systems. The nodes represent stocks in the market, while the edges represent interactions between stocks, such as synchronized price movements or mutual influences. The flow and allocation of capital resources not only reflect the structure and dynamics of the stock market, but are also profoundly influenced by investor behavior. At the same time, these behaviors are constrained by the quantity and quality of available information, which affect the effectiveness of capital allocation and hence the stability of the equity market system. In this paper, the systematic risk entropy is calculated using the following method:

When researching the entropy of systemic risk, the assignment of weight *ω* is typically based on the connectivity among nodes (i.e., shares) within the network. Building on the preceding context, mutual information is employed as a metric to gauge the correlation between stocks, and the edge weights are calculated based on the mutual information values between stocks. This approach can be regarded as a measure of the weight between pairs of stocks. At this juncture, the systemic risk entropy of the entire network is calculated as follows:

(13) $$H=-\sum_{i=1}^{N}\sum_{j=1,j\neq i}^{N}p(i,j)\cdot I(i;j)\cdot \log\left[p(i,j)\right]$$

Within the model, *P*(*i*, *j*) signifies the joint probability distribution of stocks *i* and *j*, and *I*(*i*; *j*) is the mutual information between share *i* and share *j*, employed as the weight *ω_ij_*. The inner sum compiles the cumulative contributions of correlations for a given equity *i* with respect to other stocks *j*. Moreover, the equation presupposes that the inter-stock correlations are static. After data processing and insertion into the formula, the computed systemic risk entropy within the complex system of the equity marketplace, representing China’s significant industries within the study period, amounts to 13,975.7805.

## 3. Results and Discussion

### 3.1. Determination of Threshold

Incorporating the normal distribution characteristics exhibited in Figure 1 previously, the probability of values outside the range of [μ − 3σ, μ + 3σ] is exceptionally low. Thus, according to the 3σ rule, an effective threshold range of [0.039, 0.327] can be established, designated as the effective threshold range for the complex network of stocks.

**Figure 2:**
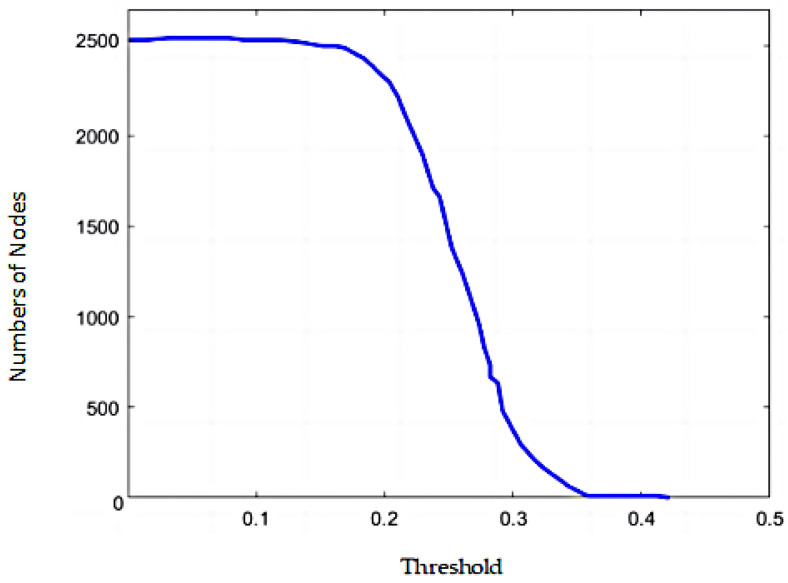
Changes in the number of nodes in the largest connected subgraph based on mutual information.

As shown in Figure 2, further analysis of the largest connected subgraph reveals that when the threshold is set to 0.160, the amount of points in the largest connected subgraph begins to decline sharply, stabilizing only when the threshold reaches 0.362. Therefore, the optimal threshold range for the complex network of financial stocks, after threshold optimization based on mutual information, is determined to be [0.160, 0.327]. When selecting the threshold range, it is essential to ensure that this range can effectively identify the presence of disconnections within the complicated web of financial shares. Consequently, the value immediately preceding the onset of disconnections in the complicated network of financial stocks, as the threshold changes, is determined to be the threshold; thus, the threshold for the complicated network of financial shares constructed using the mutual information threshold method is established at 0.198.

### 3.2. Construction of the Complicated Network of the Equity Marketplace

**Figure 3:**
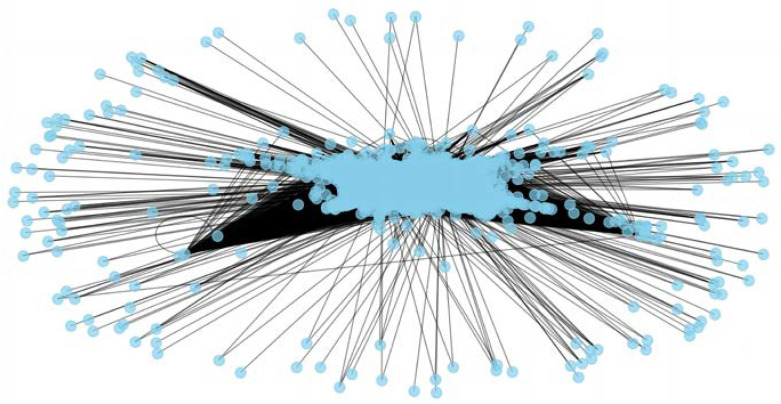
The complex network of the equity market based on mutual information.

For the complex network of the financial stock market based on a mutual information threshold, setting the threshold at 0.198 eliminates all values below 0.198 while retaining those above it. These data are then transformed into a mutual information matrix and visualized using Python 3.12, resulting in the intricate network of the financial stock market depicted in Figure 3.

### 3.3. Characteristics of the Intricate Network of the Equity Market

(1) Small-world properties

To investigate whether the financial stocks network constructed using the mutual information threshold method exhibits small-world properties, we reference the study by Watts and Strogatz [27]. A random network with the same number of nodes as the financial stock network was generated. This random network provided corresponding clustering coefficients and average shortest path lengths, allowing for a comparative analysis to substantiate the existence of small-world properties in the financial stock network.

From Table 1, it can be concluded that although the average shortest path of the intricate network of the financial share market constructed in this article is not significantly different from that of a stochastic grid, its clustering coefficient is six times that of the random network, indicating the presence of small-world properties.

(2) Scale-free properties

**Figure 4:**
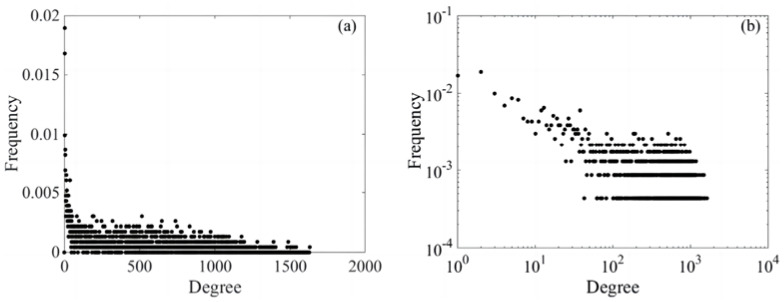
Degree distribution of the points in the complex network of the financial share marketplace; (**a**) shows that when the threshold is 0.198, the degree distribution of nodes in the complex network of financial stocks follows the long tail distribution, and (**b**) further investigates whether the network satisfies the power law distribution by using the log-log coordinates.

Figure 4a shows the degree distribution of junctions in the complex network of financial shares at a threshold of 0.198. The degree distribution follows a long-tail distribution, better reflecting the probability changes in nodes with smaller degrees. Figure 4b depicts the degree distribution of nodes within the complex network of financial stocks at a threshold of 0.198, presented on a double logarithmic scale. In Figure 4b, the data points in the high-degree region (right side) are distributed along a roughly linear trend, which corresponds to the characteristics of a power-law distribution. In the low-degree region (left side), the distribution of data points significantly deviates from a straight line. This indicates that the distribution does not conform to the power-law hypothesis in the low-degree region. The research results indicate that this complex network cannot be strictly classified as a scale-free network.

### 3.4. Key Nodes in the Complicated Network of the Equity Market

During the construction of the complex network of financial stocks, the importance of each node within the entire network is assessed by analyzing its degrees. Using Python software, the degrees of dots within the complex network of financial stocks are calculated and ranked in descending order. The top 10 nodes with the highest degrees are selected, as shown in Table 2.

The data in the table indicate that the node represented by Shandong Haihua has the closest connections with other nodes, reaching 1958, followed by Changhong Huayi from the utility industry, with a degree of 1875. Overall, the industrial sector accounts for 30% of the top ten nodes ranked by degree in the network constructed based on the mutual information threshold. The pharmaceutical healthcare, utilities, and transportation sectors each account for 20% of the top ten nodes, while the media information services sector only makes up 10%. However, all ten nodes occupy important positions within the entire network.

In complex networks, basic centrality metrics used to measure the importance of points in undirected networks include degree centrality, betweenness centrality, and closeness centrality. Betweenness centrality refers to the proportion of edges between two dots in relation to the entire mesh; a higher betweenness centrality indicates a larger proportion of such edges, often found in networks of the same type. When nodes in a network are closer to each other, their betweenness centrality increases. Closeness centrality refers to the proportion of edges between a node and other nodes in the network; a higher closeness centrality indicates a larger proportion of such edges. Based on this, the betweenness and closeness centralities of all nodes in the complex network of financial stocks were calculated, again selecting the top 10 nodes for focused analysis. The results are presented in Table 3.

It is evident that the industrial sector, represented by Ningbo Thermal Power, continues to stand out in terms of betweenness and closeness centrality, influencing and controlling 82.1% of the information and risk transmission within this complex network. In the key nodes based on degree and betweenness centrality, the media information services, pharmaceutical healthcare, transportation, and utility sectors all hold significant positions, making these four sectors the most important types of nodes within the stock complex network. The industrial sector accounts for 30% of the key nodes based on both degree and centrality, with media information services at 10%. In the key nodes based on closeness centrality, the transportation sector accounts for 10%, indicating that these two sectors occupy a smaller proportion compared to other industries.

### 3.5. Stability of the Intricate Network of Stocks

This section, based on nodes removal, explores the relationship between the ratio of nodes in the largest connected subgraph (S) and the scale of removed nodes (F), resulting in the system stability measurement illustrated below. The horizontal axis F represents the proportion of nodes that are attacked or removed. When F = 0, no nodes are removed; when F = 1, all nodes are removed. The vertical axis S represents the proportion of the largest connected subgraph among the remaining nodes for a given F value. When S = 1, all nodes are connected; when S = 0, there are no connected nodes, indicating a complete disconnection.

**Figure 5:**
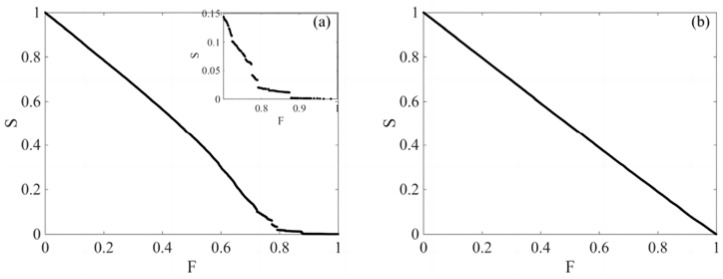
The trend of change in the proportion of nodes in the largest connected subgraph under intentional attack and random attacks; (**a**) shows a deliberate attack on critical nodes. When F approaches 1, the network connectivity decreases sharply. (**b**) represents the random selection of nodes for attack and observes the changes in connectivity. Deliberate attacks are more destructive to the largest connected subgraph.

Figure 5, respectively, depicts the trend of change in the proportion S of nodes in the largest connected subgraph to the total number of nodes in the original network under intentional and random attack strategies within the complex network of financial stocks, as a function of the proportion F of removed nodes. The graphs indicate that under an intentional attack strategy, S tends to zero when F is less than 0.87; under a random attack strategy, S decreases on a macro level as F increases, and as F approaches 1, the proportion of nodes in the largest connected subgraph, S, tends towards 0, showing the typical scale-free characteristics of the complex network of financial stocks.

### 3.6. Stability of the Equity Market Intricate Network at High and Low Thresholds

#### 3.6.1. Stability Analysis of the Share Market Complex System at a High Threshold

In the calculations described above, an optimal threshold range of [0.160, 0.327] is determined. Under these conditions, following the threshold method’s requirements, all mutual information values below 0.327 are eliminated, retaining only those above 0.327. The remaining figures are then converted into a mutual information matrix, resulting in the high-threshold complex network of the financial stock market as shown below.

**Figure 6:**
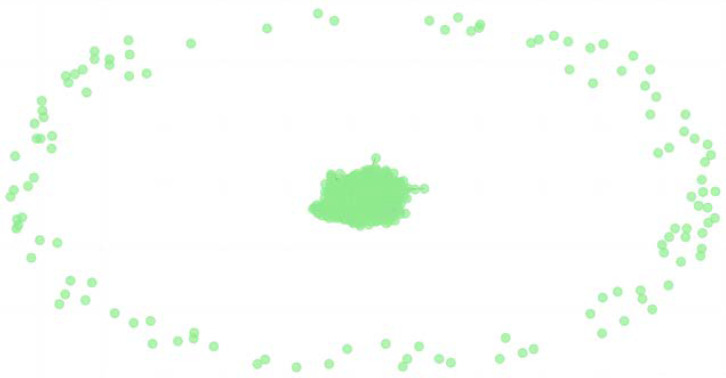
High-threshold complex web of the financial stock market.

As depicted in Figure 6, in the high-threshold network, most nodes exhibit high clustering, forming a large connected subgraph. This indicates that nodes within this area share more information and are highly interconnected. When central nodes are attacked, other nodes connected to this part are also affected. However, a small portion of peripheral nodes remains isolated due to the high threshold value limiting network connectivity. These disconnected nodes are unaffected by the central nodes and play a minimal role in the network.

**Figure 7:**
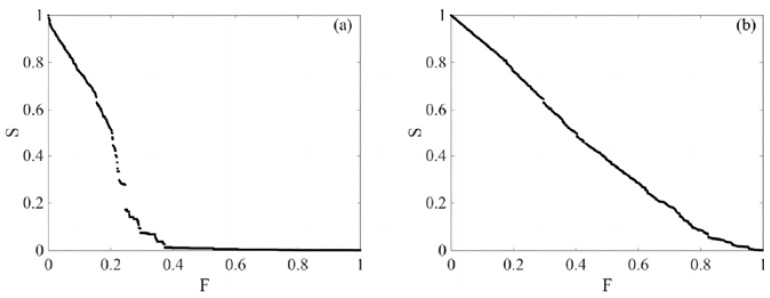
Attack scenarios on the high-threshold stock market complex network; (**a**) shows that after strengthening the standards for node importance or connectivity, under deliberate attack, S will obviously break and jump. The largest connected subgraph will suddenly split into several small subgraphs, which will lead to a sharp decline in network connectivity. (**b**) shows no obvious fracture phenomenon.

From Figure 7, it can be observed that under an intentional attack strategy, S gradually approaches 0 when F is less than 0.61, especially within the range of [0.13, 0.39], where the decrease in S is more rapid. Under a random attack strategy, S decreases macroscopically as F increases, and S tends towards 0 as F approaches 0.95, illustrating the typical scale-free characteristics of the complicated web of financial stocks.

#### 3.6.2. Stability Analysis of the Equity Market Complicated Web at a Low Threshold

**Figure 8:**
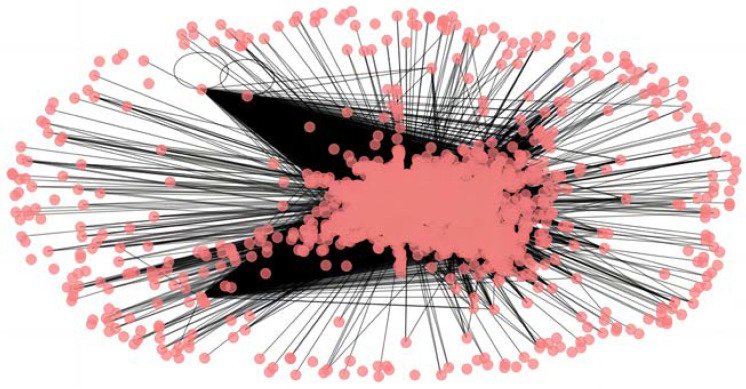
Low-threshold intricate system of the stock market.

Using the optimal threshold range of [0.150, 0.327] obtained through the calculations, setting 0.150 as a low threshold, all mutual information values below 0.150 are removed, keeping only those above 0.150. After converting the remaining figures into a mutual information matrix and visualizing it with Python software, a low-threshold intricate network of the financial share market is obtained, as shown in Figure 8.

Figure 8 reveals that most points in the network cluster together, making the entire system very dense, with only a few nodes being scattered. These scattered nodes are not significantly affected during turmoil.

**Figure 9:**
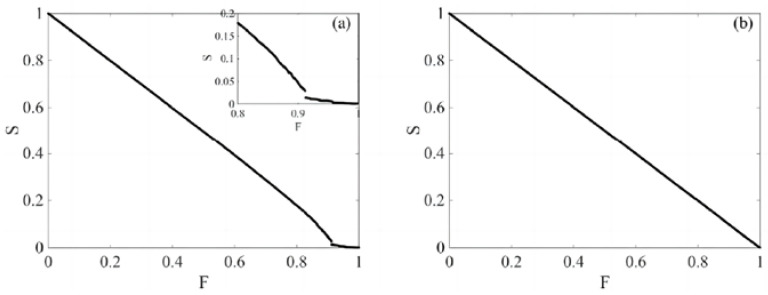
Attack scenarios on the low-threshold stock market complex network; (**a**) shows that under a low threshold, the network connectivity remains relatively stable despite the deliberate removal of nodes, and only collapses when most of the nodes are removed. The inset magnifies the details when F approaches 1, showing the final fracture. (**b**) shows the impact of random attacks on the network, where the network connectivity gradually decreases without a sudden structural change.

Figure 9 indicates that under an intentional attack strategy, S gradually tends to zero when F is less than 0.95, especially quickly within the range of [0.85, 0.912], where the decrease in S is more rapid. Under a random attack strategy, S exhibits a linear decreasing trend on a macroscopic level as F increases, and as F approaches 1, S tends towards 0.

From the above analysis, several points can be determined: First, the intricate system possesses small-world and scale-free properties, consistent with the topological features of both China’s overall equity market system and international stock market networks [28,29,30]. Second, from an industry perspective, the top ten stocks include multiple stocks from industries such as industrial, media information services, pharmaceutical healthcare, transportation, and utilities. From a corporate perspective, Changhong Huayi and Kanghui Pharmaceuticals are key nodes within the network, playing a vital role in risk control. The financial sector or financial institutions are not “at the top”. The results obtained from the above two perspectives seem to differ from those of many scholars’ research findings [31,32], which may be related to the selection of the research period. During the expansion and recovery of the pandemic, public demand for pharmaceuticals, medical care, and health services stimulated rapid industry growth, increasing their influence in the stock market. Furthermore, under an intentional attack strategy, the stock market complex network exhibits strong fragility; under a random attack strategy, the network shows greater stability. Finally, the stability of random attacks in low-threshold networks is slightly stronger than in high-threshold networks, with increased threshold values enhancing the vulnerability of networks under intentional attack strategies.

### 3.7. Analyzing Network Stability from the Perspective of Systemic Risk Entropy

**Figure 10:**
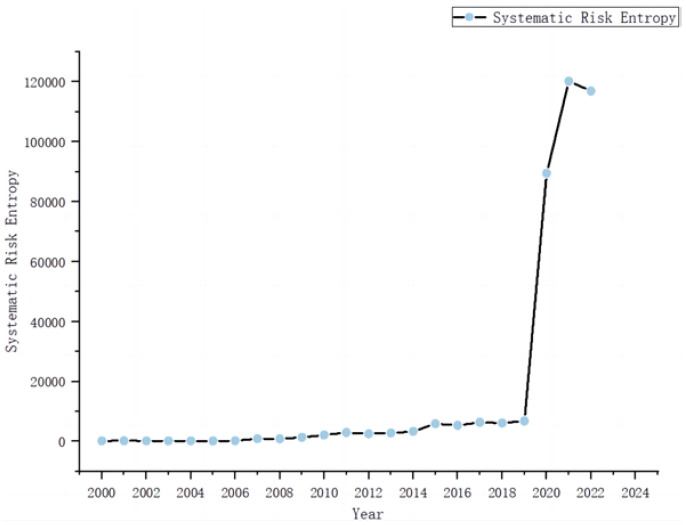
Systemic risk entropy and its variations from 2000 to 2022.

To further probe the stability of this intricate web, this section initially calculates the systemic risk entropy for each year between 2000 and 2022. Intuitively, the systemic risk entropy of the complex network within China’s major industry stock markets exhibits a trend of volatile increase, as shown in Figure 10. Compared to the year 2000, the systemic risk entropy in 2022 has increased nearly 60-fold, indicating that the instability of this complex network has become increasingly prominent.

Both the 2008 financial crisis and the recent COVID-19 pandemic are examples of exogenous shocks. However, the systemic risk entropy following the 2008 financial crisis is significantly lower compared to that during the COVID-19 pandemic. This complex phenomenon involves multiple factors, which can be primarily considered from the following two perspectives:

Firstly, regarding the nature and scope of the shocks, the financial crisis was primarily triggered by structural issues within the financial markets, such as the subprime mortgage crisis, high-leverage operations of financial institutions, and the misuse of complex financial products. Although it had a significant impact on the global economy, its effects were mainly concentrated within the financial markets and specific sectors (e.g., real estate and financial services). In contrast, the COVID-19 pandemic is a global public health crisis that has broadly affected all countries and economic sectors. It led to a significant halt in global economic activities, supply chain disruptions, and a sharp decline in consumer demand. The breadth and depth of its impact far exceed those of the financial crisis, which also explains why the systemic risk entropy during the 2008 financial crisis appears relatively moderate in the graph.

Secondly, from the perspective of market reaction and policy response, following the 2008 financial crisis, governments and central banks swiftly implemented a series of countermeasures. Post-crisis, governments and regulatory bodies worldwide strengthened financial market regulations, introducing stringent regulatory measures and raising capital adequacy and liquidity requirements for banks. These measures stabilized the markets to some extent, reduced uncertainty, and mitigated systemic risk.

**Figure 11:**
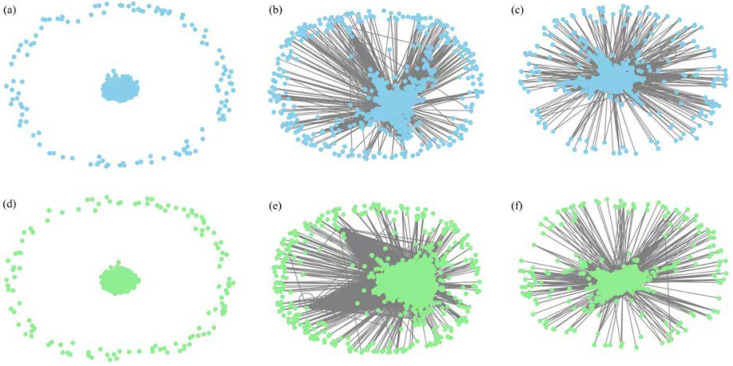
Complex networks before and after 2019 under high and low thresholds compared to the original state. (**a**) High-Threshold Network (before 2019); (**b**) Low-Threshold Network (before 2019); (**c**) Original Network (before 2019); (**d**) High-Threshold Network (after 2019); (**e**) Low-Threshold Network (after 2019); (**f**) Original Network (after 2019).

Moreover, data analysis reveals that around 2019, the entropy value experienced its most significant fluctuations, thereby serving as a demarcation point. The changes are illustrated in Figure 11. This prompted the construction of complex networks for two distinct periods: 1990–2019 and 2019–2022, under both high- and low-threshold conditions. A longitudinal comparison reveals no substantial changes in the structure of the high-threshold network around 2019. The vertical comparison reveals that the structure of the high-threshold network before and after 2019 did not change significantly. This reflects both the tightest market relationships and the most influential stock pairs. It also implies that during stress events, this could lead to a “herd effect”, exacerbating market volatility. It shows that under stress, the market may transmit risk through multiple pathways. In the low-threshold network after 2019, the connections between nodes became more dense, the network connectivity increased significantly, and the central nodes became densely connected (central nodes in their respective networks became more important). This reveals broader market connections, indicating that a disturbance in one area could quickly spread throughout the network, increasing systemic risk in the market.

The equity and currency markets are inextricably linked, and the interconnections between the cryptocurrency market and the stock market can be facilitated through various mechanisms, including the transmission of market sentiments, the sharing of investment liquidity, risk management, and arbitrage opportunities. Consequently, when considering the specific conditions of the complex network in the stock market, the cryptocurrency market also warrants attention. Research conducted by S Drożdż et al. has revealed that the COVID-19 pandemic, as an exogenous shock, impacted the topological structure of the cryptocurrency market’s complex network: it prompted a shift from a centralized to a decentralized form in the short to medium term, although this change was less pronounced on a longer-term scale. In contrast, shifts in the stock market state, such as the transition from a bull to a bear market, have visibly affected the topological structure across all scales [33].

From the perspective of stability, the impact of the COVID-19 pandemic has enhanced the robustness of the complex network in the cryptocurrency market in the short to medium term, though its effect is less significant in the long term.

It is worth discussing that transformations and feedback loops may exist between endogenous and exogenous shocks, where one type of shock triggers a response of another type, which in turn enhances or alters the initial shock. For instance, in the financial network, a global financial crisis (an exogenous shock) might lead to tightening credit markets and a loss of trust among banks (endogenous responses), further deepening the impact of the initial shock. Alternatively, the spread of information within the network (an endogenous shock) might provoke changes in public opinion, which could prompt the government to implement new policy measures (an exogenous shock), thereby again influencing the flow of information and behavioral patterns within the network. The interplay of internal and external shocks makes the measures to maintain and enhance network stability more complex.

## 4. Conclusions

Complexity science provides theoretical tools for exploring the intrinsic laws of financial markets [34], and the dynamic characteristics exhibited by various complex systems bear a high degree of similarity to the features observed in actual financial marketplaces. This similarity lays the factual foundation for the introduction of complexity science into financial markets [35]. The complex network of this stock market exhibits striking similarities with other distinctly different networks, such as small-world networks, corroborating the viewpoints of Kwapień J and Drożdż S [36].

The identification of key nodes within the network reveals that companies with a strong risk of contagion, namely those occupying significant positions within the entire network such as Changhong Huayi, Ningbo Thermal Power, and other companies ranked in the top ten by degree and centrality, can influence the entire stock market and even the broader financial market if their stock prices undergo dramatic fluctuations. For listed companies, issuing stocks for financing is merely a phase in achieving development goals. For sustainable development, on one hand, they should pay attention to the industry dynamics of companies associated with them while strengthening risk prevention, clarifying risk management objectives, and selecting appropriate risk management techniques and measures based on their characteristics to stabilize the company’s stock price. On the other hand, the government should continue to control the environmental assessment pressure on local government officials to urge companies to improve their ESG performance and reduce operational risks, implementing subsidy policies for outstanding enterprises [37,38]. For investors, it is advisable to consider the risk propagation rules of the share marketplace cautiously, make reasonable estimates of the correlation between different industries and their stocks, and adopt a diversified investment strategy to reduce losses.

Network stability analysis exhibits that this network is robust to random aggressive behavior, indicating that certain random events in the stock marketplaces of important Chinese industries do not intrinsically affect the overall price fluctuation correlation of the network. In contrast, intentional attacks can undermine the connectivity integrity of this network in short order. At the same time, however, the significant rise in systematic risk entropy suggests an incremental level of uncertainty or disorder in the share marketplace, which is a wake-up call for the stability of financial markets. In context, it is reasonable to believe that the global spread of the novel coronavirus (COVID-19), which led to extreme volatility in the global economy and financial markets in 2019–2022 and was widely linked with unfavorable factors such as declining business returns, rising unemployment, spreading investor pessimism, supply chain disruptions, and international tensions, forming a vicious cycle, is the major cause of the market’s systemic instability. The vicious circle is an important reason for the elevated systemic risk in the market.

In summary, establishing network stability analysis on the recognition of critical nodes and the entropy of systemic risk is merely a superficial and not exclusive approach and method. The crux of the issue lies in how to effectively prevent systemic risks, thereby enabling the stock market to “feed back” into the economy towards sustainable development. To this end, at the macro level, it is imperative to pay closer attention to sudden, significant events that may threaten the steadiness of the stock marketplace and to promptly implement prescient market rescue measures [39]. At the micro level, enterprises that hold significant positions within their respective sectors should make business decisions more prudently and foster a positive interaction with shareholders to better maintain the stability of the financial marketplace and, by extension, the stability of a country’s economy.

## Figures and Tables

**Table 1:** Comparison of properties between random network and equity market network.

	Random Network	Financial Stock Market Network
Clustering coefficient	0.10017	0.63178
Average shortest path	1.89992	1.92769

**Table 2:** Key nodes based on degree.

Node No.	689	1224	2634	468	1320	1247	1652	2563	1204	2812
Stock	Shandong Haihua	Changhong Huayi	Kanghui Pharmaceuticals	Hongda Hi-tech	Disen Shares	Hunan Investment	Jiangxi Guangdong Expressway	Shenzhou Taiyue	Ningbo Thermal Power	Furi Shares
Degree	1958	1875	1855	1843	1832	1712	1645	1625	1521	1268

**Table 3:** Crucial nodes based on centrality.

Rank	Betweenness Centrality			Closeness Centrality		
	Node No.	Stock	Betweenness	Node No.	Stock	Closeness
1	1125	Ningbo Thermal Power	0.821	1224	Changhong Huayi	0.00852
2	1224	Changhong Huayi	0.812	1125	Ningbo Thermal Power	0.00789
3	468	Hongda Hi-tech	0.740	1355	Jiangxi Guangdong Expressway	0.00755
4	689	Shandong Haihua	0.789	1532	Shenzhou Taiyue	0.00657
5	1320	Disen Shares	0.785	689	Shandong Haihua	0.00625
6	3254	Kanghui Pharmaceuticals	0.765	2581	Furi Shares	0.00624
7	1532	Shenzhou Taiyue	0.754	1320	Disen Shares	0.00598
8	1427	Hunan Investment	0.746	2154	Tongfeng Electronics	0.00515
9	1355	Jiangxi Guangdong Expressway	0.723	3254	Kanghui Pharmaceuticals	0.00452
10	2581	Furi Shares	0.700	468	Hongda Hi-tech	0.00324

## Data Availability

The data presented in this study are available on request from the corresponding author.
